# Risk Factors for Post-Endoscopic Submucosal Dissection Electrocoagulation Syndrome in Patients with Colorectal Neoplasms: A Multicenter, Large-Scale, Retrospective Cohort Study by the Honam Association for the Study of Intestinal Disease (HASID)

**DOI:** 10.3390/jcm13133932

**Published:** 2024-07-04

**Authors:** Hyo-Yeop Song, Seong-Jung Kim, Jun Lee, Byung Chul Jin, Dong Hyun Kim, Hyun-Soo Kim, Hyung-Hoon Oh, Young-Eun Joo, Dae-Seong Myung, Sang-Wook Kim, Geom-Seog Seo

**Affiliations:** 1 Department of Internal Medicine and Digestive Disease Research Institute, Wonkwang University School of Medicine, Iksan 54538, Republic of Korea; bfsongc@hanmail.net; 2 Department of Internal Medicine, College of Medicine, Chosun University, Gwangju 61452, Republic of Korea; ygegh@hanmail.net (S.-J.K.); leejun@med.chosun.ac.kr (J.L.); 3 Department of Internal Medicine, Research Institute of Clinical Medicine, Biomedical Research Institute, Jeonbuk National University, Jeonbuk National Medical School, Jeonju 54907, Republic of Korea; jasmintaste@gmail.com; 4 Department of Internal Medicine, Chonnam National University Medical School, Gwangju 61469, Republic of Korea; bono343@naver.com (D.H.K.); dshskim@hanmail.net (H.-S.K.); hyung1125@naver.com (H.-H.O.); yejoo@chonnam.ac.kr (Y.-E.J.); myungdaeseong@hanmail.net (D.-S.M.)

**Keywords:** colorectal neoplasms, endoscopic submucosal dissection, electrocoagulation

## Abstract

**Background and Objectives:** Colorectal endoscopic submucosal dissection (ESD) is an effective technique for removing colorectal neoplasms with large or cancerous lesions. However, there are few studies on post-ESD electrocoagulation syndrome (PECS), a complication of colorectal ESD. Therefore, this study aimed to investigate the various risk factors for PECS after colorectal ESD. **Materials and Methods:** We retrospectively analyzed the medical records of 1413 lesions from 1408 patients who underwent colorectal ESD at five tertiary hospitals between January 2015 and December 2020. We investigated the incidence and risk factors associated with PECS. Based on the data, we developed a risk-scoring model to predict the risk of PECS after colorectal ESD. **Results:** The incidence rate of PECS was 2.6% (37 patients). In multivariate analysis, the use of anti-platelet agents (odds ratio (OR), 2.474; 95% confidence interval (CI), 1.088–5.626; *p* < 0.031), a lesion larger than 6 cm (OR 3.755; 95% CI, 1.237–11.395; *p* = 0.028), a deep submucosal invasion (OR 2.579; 95% CI, 1.022–6.507; *p* = 0.045), and an ESD procedure time ≥ 60 min (OR 2.691; 95% CI, 1.302–5.560; *p* = 0.008) were independent risk factors of PECS after colorectal ESD. We developed a scoring model for predicting PECS using these four factors. As the score increased, the incidence of PECS also increased, from 1.3% to 16.6%. PECS occurred more frequently in the high-risk group (≥2) (1.8% vs. 12.4%, *p* < 0.001). **Conclusions:** In this study, the risk factors for PECS after colorectal ESD were the use of anti-platelet agents, a lesion larger than 6 cm, a deep submucosal invasion, and an ESD procedure time ≥ 60 min. The risk-scoring model developed in this study using these factors could be effective in predicting and preventing PECS.

## 1. Introduction

Colorectal endoscopic submucosal dissection (ESD) is an effective technique for removing colorectal neoplasms with large or cancerous lesions that are difficult to completely resect using endoscopic mucosal resection [1]. Colorectal ESD has the advantages of a high *en bloc* resection rate and a lower recurrence rate [2,3]. However, it presents challenges due to its technical difficulty, longer procedure time, and the increased risk of complications such as perforation and bleeding [4,5,6,7,8].

One of the complications of colorectal ESD, post-ESD electrocoagulation syndrome (PECS) is clinically diagnosed through the identification of localized abdominal pain and a fever or an inflammatory response, such as leukocytosis or the elevation of C-reactive protein levels, occurring after ESD without obvious findings of a perforation [9]. Therefore, PECS can cause significant discomfort for patients and lead to an increase in the duration of the hospital stay and in medical costs [10]. Additionally, inadequate management can lead to serious complications, including peritonitis resulting from a delayed perforation [11].

The incidence of PECS reported so far has varied widely across studies, ranging from 4.8% to 40.8% [9,12,13]. The known risk factors for PECS have also been reported to vary, and they include the tumor size, the procedure duration, the patient being of female sex, the tumor location, the presence of submucosal fibrosis, the amount of submucosal fluid, and a lack of expertise [4,5,6,7,9,10,12,13]. This is because there have been few studies on PECS to date, and most of them are small-scale retrospective studies from single centers. In particular, PECS can improve with conservative treatments alone, so its outcomes may not have been properly evaluated.

Therefore, the aim of this study was to identify the incidence of PECS, evaluate the related clinicopathological risk factors, and assess the risk of developing PECS according to the identified risk factors by analyzing our large colorectal ESD cohort. Additionally, we proposed a risk prediction model for PECS based on the results of this study. This prediction model is intended to provide a practical tool for estimating the risk of PECS before and after ESD, aiding in the selection of the optimal post-ESD management strategy.

## 2. Materials and Methods

### 2.1. Study Population and Study Design

**Figure 1:**
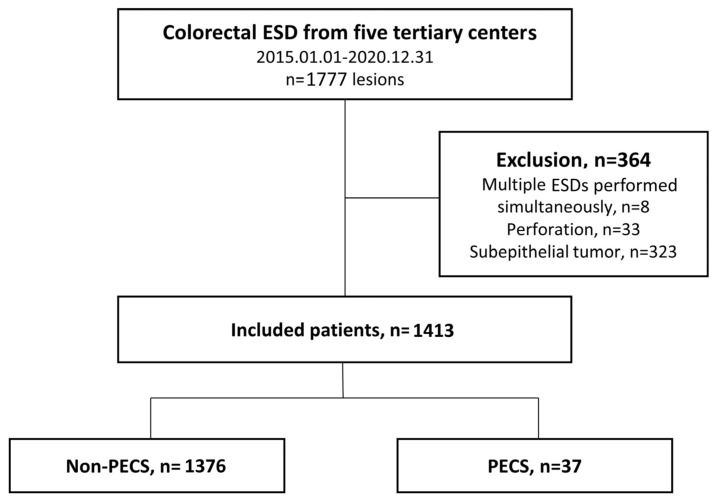
Study flowchart.

This study included 1777 colorectal neoplasms from the 1768 patients who underwent colorectal ESD between January 2015 and December 2020 at five tertiary hospitals in South Korea. The patients’ medical records were collected and analyzed retrospectively from March 2022 to July 2023 (Figure 1). The colorectal ESDs were performed according to the indications for ESD set by the Japanese gastroenterological endoscopy society [14]. The inclusion criteria were as follows: The lesions for which endoscopic *en bloc* resection is required include those for which *en bloc* resection with snare EMR is difficult to apply. Specifically, these lesions include non-granular-type laterally spreading tumors (particularly those that are pseudo-depressed), lesions showing a VI-type pit pattern, carcinomas with shallow T1 invasion, large depressed-type tumors, and large protruded-type lesions that are suspected to be carcinomas. Additionally, the inclusion criteria cover mucosal tumors with submucosal fibrosis and local residual or recurrent early carcinomas after endoscopic resection. Patients undergoing multiple ESD procedures simultaneously, those with a confirmed perforation during the ESD procedures, and those with subepithelial tumors were excluded. This study was conducted in accordance with the ethical guidelines of the Declaration of Helsinki and approved by the Institutional Review Board of the Ethics Committee of each hospital (approval numbers and dates: WKUH 2023-04-026, approved on 12 May 2023; CHOSUN 2022-03-006-001, approved on 15 March 2022; CNUH-2022-060, approved on 10 March 2022; CNUHH-2022-208, approved on 8 November 2022; and CNU 2022-12-057, approved on 9 January 2023).

### 2.2. ESD Procedure and Patient Management

**Figure 2:**
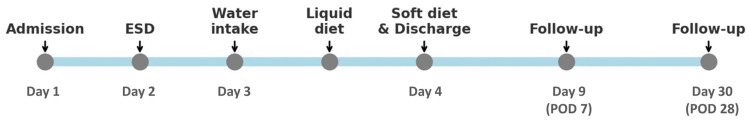
Study protocol and clinical path for colorectal ESD.

All of the patients who were scheduled to undergo a colorectal ESD were admitted to a hospital. The timing of the discontinuation and restart of anti-platelet agents before ESD was generally 5–7 days prior to the procedure to reduce the risk of bleeding. The exact timing varied depending on the type of the anti-platelet agent and the patient’s individual risk factors, and it was mostly in accordance with the American Society for Gastrointestinal Endoscopy guidelines [15,16]. For patients at a high risk of thromboembolic events, the discontinuation period was determined in consultation with the relevant departments, such as neurology and cardiology, which were responsible for the use of anti-platelet agents. The procedures were performed by seven experts who had performed >100 colorectal ESDs (S.J.K., L.J., K.S.S., S.W.K., D.H.K., D.S.M., and Y.E.J.). The choice of conscious sedation, involving a combination of midazolam, propofol, or pethidine, was at the discretion of the attending endoscopist. The ESDs were performed using CF-HQ290 or CF-H290A and CF-H260A endoscope models (Olympus, Tokyo, Japan) with a water-jet system. ESD devices, including a dual knife or insulated-tip electrosurgical knife nano (Olympus Medical Systems Corp., Tokyo, Japan) and a high-frequency surgical generator (VIO300D; ERBE, Tübingen, Germany) were utilized across all of the participating institutions. A mixture of indigo carmine, 5% fructose, and 10% glycerol in normal saline solution (Cerol; JW Pharmaceutical, Seoul, Korea or Glyfurol; Daihan Pharm Co., Ltd., Seoul, Korea) was typically used, and a mixture of indigo carmine, 0.1% epinephrine, and plasma solution, or a mixture of indigo carmine and 0.4% sodium hyaluronate (Endo-ease; UNIMED Pharm. Inc., Seoul, Korea) were used as the submucosal injection solution, depending on the hospital. After submucosal injection, a dual knife was primarily employed for mucosal incision and submucosal dissection, but an IT nano knife was additionally used depending on the operators’ preferences. If the muscle layer was damaged during the procedure, the application of clips was considered, even if there was no clear perforation. To control ESD-related bleeding, hemostatic forceps (Coagrasper^®^ FD-411UR, Olympus) were used. All of the patients who underwent an ESD had chest and abdominal X-rays immediately after the procedure to check for the presence of free air. Prophylactic antibiotics were not administered, and laboratory tests and X-rays were performed on the first day post-procedure. Patients who were diagnosed with PECS were managed using the cessation of oral intake, parenteral nutrition, and an intravenous antibiotic therapy. The absence of fever and the disappearance of abdominal tenderness were judged as a recovery from PECS, and fasting was discontinued. If a delayed perforation was suspected, an abdominal computed tomography was performed. Patients without symptoms post-ESD were allowed to drink water 24 h after the procedure and began a liquid diet at their next meal. Those patients without complications were discharged two days after the procedure. After discharge, the patients returned for follow-up visits one week and one month later to check their condition. They were instructed to contact us by phone if they experienced symptoms such as abdominal pain or hematochezia (Figure 2).

### 2.3. Data Collection and Definitions

We collected data on various factors, including the patients’ age and sex, comorbidities, antithrombotic agents, tumor size, tumor location, tumor morphology, histopathology, procedure time, the presence of submucosal fibrosis, a non-lifting sign, the use of a knife, the *en bloc* resection rate, the presence of intra-procedure bleeding, and the prophylactic coagulation status. We also assessed the clinical outcomes, such as the hospitalization day, delayed bleeding, delayed perforation, and recurrence.

We defined PECS as the presence of localized abdominal pain and a body temperature ≥ 37.8 °C, or a positive inflammatory response (white blood cell count ≥ 10,000/mm^3^ or serum CRP level ≥ 0.5 mg/dL) without obvious findings of a perforation, which developed >6 h post-ESD. To exclude abdominal pain that was due to the effect of residual gas after the procedure, PECS was defined as a case in which symptoms occurred after 6 h [9,10,17,18].

The tumor location was classified as the right colon (cecum, ascending colon, and transverse colon), left colon (descending colon and sigmoid colon), or rectum. The tumor morphology was classified as protruding lesions (0-Is/0-IIa/0-Is + IIa) and flat or depressed lesions (0-IIb/0-IIa + IIc/IIc), according to the Paris classification. A deep submucosal invasion was defined as a case in which the depth of the colorectal carcinoma’s submucosal invasion measured >1000 μm at the vertical level in the pathology report. The ESD procedure time was assessed as the time spanning from the initial submucosal injection to the completion of the submucosal dissection. A submucosal fibrosis was defined as a poor lifting of the submucosal layer and the presence of web-like fibers [19]. Bleeding treated with hemostatic forceps during the procedure was classified as intra-procedure bleeding. A post-ESD prophylactic coagulation was defined as the use of hemostatic forceps to coagulate visible vessels after the dissection was completed.

### 2.4. Statistical Analyses

Data were analyzed using SPSS software version 27.0 (IBM Corp., Armonk, NY, USA). Continuous variables were presented as mean ± standard deviation, employing the Mann–Whitney U test, whereas categorical variables were presented as frequencies and percentages, employing the Chi-square test or Fisher’s exact test. The risk factors for PECS were evaluated through a multivariate logistic regression model, with the risk factors presented as odds ratios (ORs) alongside 95% confidence intervals (CIs). The statistical significance was set at *p* < 0.05.

We developed the scoring model by assigning weights to each of the risk factors according to the β-coefficient derived from the multivariate logistic regression model of the cohort [20]. To assess the model’s goodness of fit, we employed the Hosmer–Lemeshow test. The scoring model’s performance was evaluated by determining the area under the receiver operating characteristic (ROC) curve, along with the 95% confidence interval. The optimal cutoff value distinguishing the high-risk and low-risk groups was determined using the maximum Youden index. The proportions of patients with PECS were compared using either the chi-square test or Fisher’s exact test.

## 3. Results

### 3.1. Comparison of Baseline Clinicopathological Characteristics and Therapeutic Outcomes of Colorectal ESD According to PECS

A total of 1413 lesions from 1408 patients were included in this analysis, with 364 patients excluded due to undergoing simultaneous multiple ESD procedures (n = 8), a perforation confirmed during ESD (n = 33), and the presence of subepithelial tumors (n = 323). In this study, PECS occurred in 37 patients (2.6%) (Figure 1). The baseline characteristics of the patients and their lesions are presented in Table 1. Their mean age was 65.29 ± 11.168 years, and there were 564 females (39.9%). There were no differences in age, sex, or comorbidities between the PECS and non-PECS groups, but the PECS group had a higher use of anti-platelet agents (11.6% vs. 26.3%, *p* = 0.018). Regarding the tumor characteristics, there were no differences in the tumor location or the gross finding between the two groups, but significant differences were observed in cases with tumors larger than 6 cm (2.6% vs. 13.5%, *p* < 0.001) or deep submucosal invasions (6.8% vs. 18.9%, *p* = 0.016). Regarding the ESD procedure, there were significant differences between the two groups in procedure time ≥ 60 min (16.6% vs. 43.2%, *p* < 0.001), a non-lifting sign status (27.9% vs. 40.5%, *p* = 0.042), and the occurrence of intra-procedure bleeding (23.8% vs. 40.5%, *p* = 0.019); there was no difference between the two groups in the rate of post-ESD prophylactic coagulation (98.7% vs. 100%, *p* = 1.000).

### 3.2. The Risk Factors for PECS Related to Colorectal ESD

In the multivariate analysis using logistic regression, the independent risk factors for PECS after colorectal ESD were the use of anti-platelet agents (odds ratio (OR), 2.474; 95% confidence interval (CI), 1.088–5.626; *p* < 0.031), a lesion larger than 6 cm (OR 3.755; 95% CI, 1.237–11.395; *p* = 0.028), deep submucosal invasive cancer (OR 2.579; 95% CI, 1.022–6.507; *p* = 0.045), and an ESD procedure time ≥ 60 min (OR 2.691; 95% CI, 1.302–5.560; *p* = 0.008) (Table 2).

### 3.3. Clinical Outcome and Prognosis According to PECS

The PECS group had more fasting days (1.28 vs. 2.05 days, *p* < 0.001) and longer hospital stays (4.33 vs. 6.38 days, *p* < 0.001) than the non-PECS group. Delayed bleeding occurred more frequently in the PECS group (2.3% vs. 8.1%, *p* = 0.047), but no cases of delayed perforation were observed. There was no difference in recurrence between the two groups (1.3% vs. 2.7%, *p* = 0.487), and there were no deaths (Table 3).

### 3.4. Risk-Scoring Model for Predicting PECS

**Figure 3:**
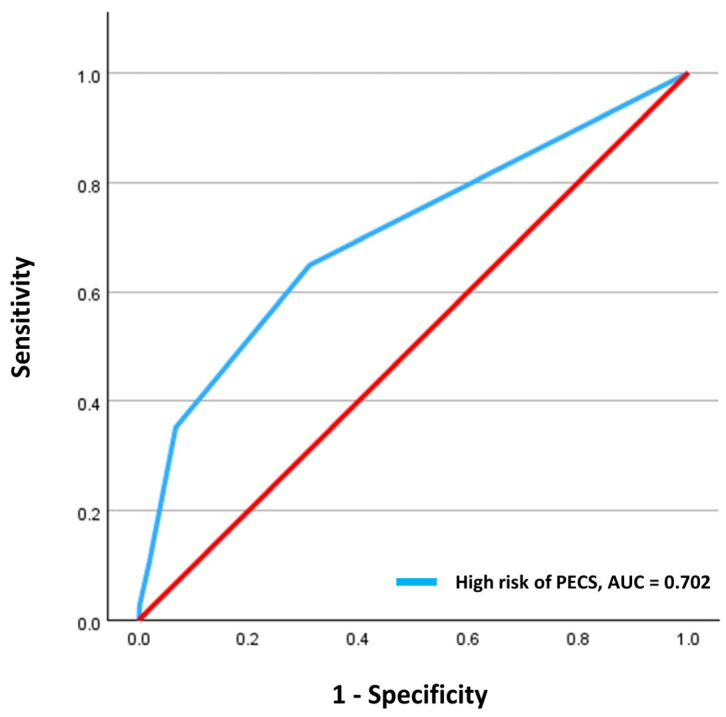
The discrimination performance of the model was assessed using receiver operating characteristic curves.

A scoring model to predict the risk of PECS after colorectal ESD was developed by assigning risk score points to each risk factor. Each risk factor was assigned one point based on the β-coefficient from the multivariate analysis. The area under the ROC curve for the risk-scoring model was 0.702 (95% CI, 0.605–0.799), indicating a good discrimination ability and the Hosmer–Lemeshow results (χ^2^ = 1.630; *p* = 0.990) demonstrated a good goodness of fit for the model (Figure 3). In this cohort, the incidence of delayed bleeding increased from 1.3% to 16.6% as the risk score increased from 0 to 3. Based on a cutoff score of 2, patients were divided into low-risk (n = 1308, 92.6%) and high-risk (n = 105, 7.4%) groups. PECS occurred significantly more frequently in the high-risk group than in the low-risk group (1.8% vs. 12.4%, *p* < 0.001). The sensitivity, specificity, positive predictive value, and negative predictive value for the occurrence of PECS in the high-risk group were 35.1%, 93.3%, 13.1%, and 98.1%, respectively (Table 4).

## 4. Discussion

This study, which included a large sample of 1413 lesions, found that the incidence of PECS was 2.6%. The independent risk factors for PECS after colorectal ESD were identified as the use of anti-platelet agents, lesions larger than 6 cm, deep submucosal invasive cancers, and an ESD procedure time of 60 min or more.

PECS is a complication characterized by abdominal pain and an inflammatory response without overt perforation, caused by transmural burns and localized peritonitis that result from electrocoagulation damage to the intestinal wall, leading to serosal inflammation. Since most cases can be cured with conservative treatment, including antibiotics, it is important to be well aware of PECS to prevent excessive treatments such as surgery [17,21,22]. Compared to post-polypectomy coagulation syndrome following EMR, the incidence of PECS has been reported to be much higher, ranging from 4.8% to 40.8% across studies [4,5,6,7,9,10,12,13]. This is associated with the characteristics of the colon, which is tortuous, flexible, and has a narrow lumen and thin walls, as well as the long procedure time and the repeated electrocautery involved in ESD.

However, in the present study, the incidence of PECS was 2.6%, which is lower than those in the previously reported studies [9,12,13]. The incidence of PECS in previous studies, which included both endoscopists and trainees, may have been higher due to the volume of ESDs performed annually and the proficiency of the endoscopists affecting the occurrence of ESD complications and the procedural outcomes [2,23]. The present study only involved experts with experience of more than 100 cases of colorectal ESD. In addition, the present study targeted patients who were treated between 2015 and 2020, and compared with previous studies, the development of endoscopic equipment, such as knives, caps, and colonoscopes, the increase in the use of carbon dioxide gas, and the development of ESD technology may have led to the low incidence of PECS in the present study. Considering the aforementioned factors, the low incidence of PECS in this study can be reasonably inferred to be a result of the increasing number of experts in colorectal ESD, an improved awareness of PECS, and the advancements in ESD technology.

A noteworthy point in this study was that the use of anti-platelet agents is a risk factor for PECS after colorectal ESD. The mechanism by which anti-platelet agents influence PECS is unclear. But anti-platelet agents may increase the risk of bleeding during a colorectal ESD procedure, necessitating the use of electrocautery for hemostasis, which can lead to thermal injury. Additionally, patients on anti-platelet therapy often have underlying conditions such as diabetes, hypertension, metabolic syndrome, stroke, or myocardial infarction, which are linked to microcirculatory dysfunction. Impaired microcirculation can hinder the healing of inflammation post-procedure, thereby promoting the development of PECS [24].

As in previous studies [10,13,18,19,22,23,25], this study also found that a procedure time ≥ 60 min, a deep submucosal invasion, and lesions larger than 6 cm were risk factors for PECS after colorectal ESD. Generally, when the ESD procedure time is prolonged or the lesion size is large, increasing the frequency of electrocoagulation, the risk of developing PECS may be higher [26]. Omori et al. reported that an ESD procedure time ≥ 90 min (OR 2.664, 95% CI 1.053–6.742, *p* = 0.039) was a risk factor [22]. Yamamoto et al. also reported that an ESD procedure time ≥ 60 min (OR 4.14, 95% CI 1.78–9.60, *p* = 0.0009) was a risk factor for PECS [25]. Additionally, several previous studies [10,13,18,22], including the research by Kim et al., have identified a lesion size of 4 cm or larger as a risk factor for PECS. In several previous studies [19,23], a deep submucosal invasion was reported to be associated with severe fibrosis, long ESD procedure times, incomplete resection, and additional surgery. This association increases the difficulty of ESD procedures and the incidence of PECS.

Meanwhile, prophylactic coagulation was found to have no significant association with the occurrence of PECS. While additional coagulation after submucosal dissection was hypothesized to increase PECS incidence due to further thermal damage, our study found no such link. Japanese guidelines recommend minimal prophylactic coagulation, including exposed vessels [27]. However, in this study, comprehensive prophylactic coagulation was frequently performed on the mucosal defect areas following ESD. Prophylactic coagulation typically involves contact coagulation using hemostatic forceps or knife tips, which may not cause deep-layer damage sufficient to induce PECS. Therefore, the use of superficial electrocautery likely did not significantly impact PECS development. Further research is necessary to confirm these findings.

In this study, most of the patients with PECS recovered well without complications, but the duration of fasting and the length of hospital stay were significantly prolonged. In particular, patients with PECS experienced a higher incidence of delayed bleeding. The relationship between PECS and delayed bleeding has not yet been confirmed. The known risk factors for delayed bleeding after colorectal ESD include the rectosigmoid location, the lesion size, the use of anti-platelet agents, and significant intra-procedure bleeding events [28,29]. Considering that some of the risk factors for delayed bleeding overlap with those for PECS, it is plausible that the thermal injury and the inflammatory response causing PECS may also contribute to delayed bleeding. Although the use of prophylactic antibiotics and endoscopic clipping closure were thought to be effective in preventing PECS, they were ineffective in previous studies [17,22,24,29]. Therefore, it is important to accurately identify and predict the high-risk groups for PECS, promptly detect PECS, and provide immediate treatment.

Therefore, we have developed a risk-scoring model for predicting PECS following colorectal ESD, reflecting the results of this study. When each of the four risk factors was assigned a value of 1 point and patients with a score of 2 or more were classified as high-risk, the model demonstrated quite good discriminative ability. Our PECS prediction score has the potential to significantly impact clinical practice. Although the generalization of this PECS risk prediction model through external validation is necessary, if patients have the aforementioned risk factors for PECS, especially if they have two or more risk factors, the probability of PECS occurring according to the prediction model exceeds 10%. Therefore, special attention should be paid to these high-risk patients to prevent PECS. During the ESD procedure, minimizing the use of electrocoagulation, carefully managing coagulation, and implementing prophylactic clipping should be considered. After the procedure, patients should be closely monitored for the occurrence of PECS, and immediate treatment should be administered if PECS develops.

The present study had several limitations. First, because this was a multicenter retrospective study, selection and information biases may be present. Second, because this was a multicenter study, the ESD procedures, such as the electrosurgical unit setting and submucosal injection solution, differed at each institution, which may have affected the results. Third, we cannot rule out the possibility of micro-perforation because not all of the patients underwent computed tomography. Finally, the PECS risk prediction model proposed in this study has not been externally validated. To ensure the model’s generalizability, additional external validation is essential.

## 5. Conclusions

This multicenter, large-scale, retrospective cohort study revealed that the use of anti-platelet agents, a lesion size ≥ 6 cm, a deep submucosal invasion, and an ESD procedure time ≥ 60 min were significant independent risk factors for PECS after colorectal ESD. The risk-scoring model incorporating these risk factors can predict the risk of PECS. This prediction can help in estimating the risk of PECS, planning colorectal ESD, and carefully performing the procedure to prevent PECS.

## Figures and Tables

**Table 1:** Comparison of baseline clinicopathological characteristics and therapeutic outcomes of colorectal ESD according to PECS.

	Total (n = 1413)	Non-PECS (n = 1376)	PECS (n = 37)	*p*-Value
Age (means ± SD)	65.29 ± 11.168	65.3 ± 11.187	65 ± 10.608	0.753
≥65 (n, %)	707 (52.0)	691 (52.2)	16 (45.7)	0.496
Sex (Female, n, %)	564 (39.9)	549 (97.3)	15 (2.7)	1.000
BMI (n, %)	24.23 ± 3.38	24.24 ± 3.38	23.88 ± 3.57	0.338
Hypertension (n, %)	565 (39.9)	549 (39.9)	16 (43.2)	0.748
Diabetes mellitus (n, %)	279 (19.7)	269 (19.5)	10 (27.0)	0.260
CKD (n, %)	25 (1.8)	25 (1.8)	0	1.000
Anti-platelet agents (n, %)	168 (11.9)	159 (11.6)	9 (24.3)	0.018
Anticoagulants (n, %)	13 (1)	12 (1)	1 (2.7)	0.293
ASA (≤2, n, %)	674 (47.7)	659 (47.9)	15 (40.5)	0.377
Lesion with size (means ± SD, cm)	2.95 ± 1.26	2.93 ± 1.19	3.76 ± 1.96	<0.001
Lesion larger than 6 cm (n, %)	41 (2.9)	36 (2.6)	5 (13.5)	<0.001
Lesion location (n, %)				0.583
Right colon	713 (50.5)	697 (50.7)	16 (43.2)	
Left colon	329 (23.3)	318 (23.1)	11 (29.7)	
Rectum	371 (26.3)	361 (26.2)	10 (27.0)	
Gross finding (n, %)				0.973
0-Is/0-IIa/0-Is + IIa	1066 (75.4)	1038 (75.4)	28 (75.7)	
0-IIc/0-IIa + IIc/scar	347 (24.6)	338 (24.6)	9 (24.3)	
Histopathology (n, %)				0.016
Adenoma	1051 (74.4)	1028 (74.7)	23 (62.2)	
Superficial SM invasion cancer	262 (18.5)	255 (18.5)	7 (18.9)	
Deep SM invasion cancer	100 (7.1)	93 (6.8)	7 (18.9)	
ESD procedure time (means ± SD, minutes)	38.82 ± 34.18	38.24 ± 33.28	60.41 ± 55.18	0.010
≥60 min	244 (17.2)	228 (16.6)	16 (43.2)	<0.001
Submucosal fibrosis (n, %)	455 (32.2)	438 (31.8)	17 (45.9)	0.054
Non-lifting sign (n, %)	399 (28.2)	384 (27.9)	15 (40.5)	0.042
Use of more than one knife (n, %)	63 (4.5)	60 (4.4)	3 (8.1)	0.226
*En bloc* resection (n, %)	1268 (89.7)	1237 (89.9)	31 (83.8)	0.226
Intra-procedure bleeding	340 (24.1)	328 (23.8)	15 (40.5)	0.019
Prophylactic coagulation	1395 (98.7)	1358 (98.7)	37 (100.0)	1.000

PECS, post-endoscopic submucosal dissection electrocoagulation syndrome; ESD, endoscopic submucosal dissection; BMI, body mass index; CKD, chronic kidney disease; ASA, American Society of Anesthesiologists score; CCI, Charlson Comorbidity Index; SM, submucosal.

**Table 2:** The risk factors for PECS related to colorectal ESD.

Risk Factors	β	Odd Ratio	95% Confidential Interval	*p*-Value
Anti-platelet agents	0.906	2.474	1.088–5.626	0.031
Lesion with size (≥6 cm)	1.323	3.755	1.237–11.395	0.028
Deep submucosal invasion	0.947	2.579	1.022–6.507	0.045
ESD procedure time (≥60 min)	0.990	2.691	1.302–5.560	0.008
Submucosal fibrosis (n, %)	0.298	1.347	0.384–4.724	0.642
Non-lifting sign (n, %)	−0.038	0.962	0.265–3.495	0.953
Intra-procedure bleeding	0.553	1.738	0.870–3.475	0.118

PECS, post-endoscopic submucosal dissection electrocoagulation syndrome; ESD, endoscopic submucosal dissection.

**Table 3:** Clinical outcomes and prognoses according to PECS after colorectal ESD.

	Non-PECS (n = 1376)	PECS (n = 37)	*p*-Value
Fasting days (mean, range)	1.28 (0–5)	2.05 (1–7)	<0.001
Hospitalization days (mean, range)	4.33 (2–38)	6.38 (2–31)	<0.001
Delayed bleeding (n, %)	31 (2.3)	3 (8.1)	0.047
Delayed perforation (n, %)	0	0	
Recurrence (n, %)	18 (1.3)	1 (2.7)	0.487
Death (n, %)	0	0	
Fasting days (mean, range)	1.28 (0–5)	2.05 (1–7)	<0.001

PECS, post-endoscopic submucosal dissection electrocoagulation syndrome; ESD, endoscopic submucosal dissection.

**Table 4:** Risk-scoring model for predicting PECS after colorectal ESD.

Risk of PECS	Incidence of PECS	Sensitivity	Specificity	PPV
Low risk (0–1)	1.8% (24/1308)			
0	1.3% (13/961)			
1	5.3% (24/452)			
High risk (2–3)	12.4% (13/105)	35.1%	93.3%	13.1%
2	13.0% (12/92)			
3	16.6% (1/6)			

PECS, post-endoscopic submucosal dissection electrocoagulation syndrome; ESD, endoscopic submucosal dissection; PPV, positive predictive value; NPV, negative predictive value.

## Data Availability

The data are not publicly available due to privacy and ethical restrictions. Data presented in this study are available upon request from the corresponding author.
